# Multispecies Assessment of Anthropogenic Particle Ingestion in a Marine Protected Area

**DOI:** 10.3390/biology11101375

**Published:** 2022-09-20

**Authors:** Montserrat Compa, Carme Alomar, María Francesca López Cortès, Beatriz Rios-Fuster, Mercè Morató, Xavier Capó, Valentina Fagiano, Salud Deudero

**Affiliations:** 1 Centro Oceanográfico de Baleares (IEO-CSIC), Muelle de Poniente s/n, 07015 Palma, Spain; 2 Dirección General de Espacios Naturales y Biodiversidad, Parque Nacional Marítimo-Terrestre del Archipiélago de Cabrera, Gremi de Corredors 10, Polígon de Son Rossinyol, 07009 Palma, Spain

**Keywords:** marine diversity, marine plastic, ingestion, marine habitats, Mediterranean Sea

## Abstract

**Simple Summary:**

Plastic pollution presents a growing concern for marine biodiversity, and among these, anthropogenic particles (APs) are entering marine ecosystems at an alarming rate from land and sea sources. In this study, we quantify the ingestion of APs across fish, sea urchins, sea cucumbers (echinoderms), bivalves (molluscs), and jellyfish (cnidarians), identify biotic and abiotic factors that might influence the ingested items; and identify ingestion patterns based on taxonomic groups, trophic guilds, and habitats. Ingestion of APs was observed in the majority of the species analyses with occurrence ranging from 0% to 100%. The study results indicate that plastic pollution poses a threat to species found within the marine protected area of the Cabrera Marine-Terrestrial National Park despite their protection status. The multispecies approach provided a better understanding of the high level of AP exposure to species, highlighting the associated damage to marine biodiversity related to marine waste.

**Abstract:**

We have applied a multispecies ecosystem approach to analyse the ingestion of anthropogenic particles (AP) in the gastrointestinal tract of 313 individuals (17 fish species and 8 invertebrate species) from pelagic, demersal and benthic habitats in a marine protected area off the Western Mediterranean (Cabrera National Park). We have quantified and characterized the ingestion at several taxonomic levels of fish, sea urchins, sea cucumbers, bivalves, and jellyfish in relation to biotic/abiotic factors based on taxonomic groups, trophic guilds (functional groups) and habitats. AP ingestion occurrence ranged from 26 to 100% with no significant differences among taxonomic groups. The fish within the MPA showed an overall ingestion occurrence ranging from 0 to 100%, the echinoderms from 29 to 100%, the bivalves from 72 to 96% and the jellyfish 36% ingestion. The ecosystem approach applied to evaluate overall AP ingestion within the species reported that for trophic guilds, the omnivorous species ingested the highest amounts of anthropogenic items, while herbivores ingested significantly fewer items than all other trophic guilds. Moreover, no significant differences were found amongst habitats, indicating a homogeneous spatial distribution of APs at all studied habitats. The multispecies approach provided insight into the high APs exposure to species within Cabrera MPA, highlighting the potential harm linked with marine litter that threatens marine biodiversity.

## 1. Introduction

Human impacts on the marine environment present a continuously growing concern, and among these, anthropogenic particles (AP), which include microplastics (<5 mm) and other particles from certified anthropogenic origins such as artificially dyed fibres and other man-made materials, are entering marine ecosystems at an alarming rate from land and sea sources [1,2]. Plastic litter is a growing concern for marine diversity, with global plastic production exceeding 368 million metric tonnes/year, of which between 4.8 and 12.7 million tons are estimated to enter the oceans annually with the records of harm these items might cause to marine fauna steadily increasing [2,3]. The overlap between the presence of plastic litter in the marine environment and its impact on species has been identified through ingestion studies across marine organisms by contrasting models and concentrations found in different compartments of the marine environment, such as on the sea surface and on the seafloor [4,5,6]. Furthermore, models of hotspot areas calculated globally and in the Mediterranean Sea showed that sea turtle species, seabirds, and other marine species are at high risk of interacting with plastic marine debris [7,8,9], an indication of the severity posed by plastic waste on marine diversity globally and at the regional scale.

Within the western Mediterranean Sea, studies have highlighted the ingestion of plastic litter in marine organisms, especially marine fish [10,11,12,13,14] and it is important to identify the relationship between the ingestion of plastic litter and trophic guilds [15]. In this study, we follow the definition of trophic guilds as detailed in [16], and aggregations of species with similar diet compositions. In addition to the potential relationships between ingestion and trophic guilds, the interaction of plastic litter with marine organisms has been shown to cause negative effects in wild organisms ranging from acute toxicity and endocrine disruption to oxidative stress, including a false sense of satiety, which can lead to malnutrition [17]. Furthermore, persistent organic pollutants (POPs) present in the marine environment, such as PCBs, PAHs and PBDEs, can cause acute toxicity if absorbed by plastic items/particles [18], while plastic additives such as plasticizers, flame retardants, colorants, and other harmful additives that are all associated with plastic packaging can cause endocrine disruption and activate antioxidant enzymes such as reduced glutathione (GSH) and the facilitation of oxidative damage [19,20]. Furthermore, increased glutathione S-transferase (GST) biomarker activity in the proposed bioindicator species *Mullus surmuletus* indicates an induction of detoxification systems following ingestion and exposure to microplastics in its natural habitat [12]. This has also been confirmed in several laboratory studies in both fish and mussels for several other enzymatic responses and biomarkers [21,22,23]. Further laboratory studies have identified that 21-day exposure to plastics was enough to trigger the activation of antioxidant enzymes in addition to behavioural effects with increased incidence of bolder social interactions in *Sparus aurata*, especially when feeding [24]. In addition to the physical and behavioural responses induced by plastic exposure, there is also increasing concern in terms of the effects on human health as a consequence of ingesting commercially important seafood species and their potential to act as vectors of contaminants during human consumption [25].

Marine protected areas (MPAs) have been widely created as a measure to minimize the impact of human activities on the marine environment. However, several MPAs around the world have been identified as sinks for accumulating marine litter, which has been found to impact the fauna inhabiting these areas [26]. Within the Mediterranean Sea, plastic litter models have reported a synergy between anthropogenic drivers and hydrodynamic transport, especially to MPAs, which can account for the high plastic flux on MPA coastlines [27]. Considering that the Mediterranean Sea is a hotspot for marine diversity and it is affected by cumulative effects of multiple human stressors, recent 3D numerical models of plastic pollution have highlighted that coastal regions of the Mediterranean Sea are at high risk of plastic exposure and that the current protection levels of MPAs, most of them at a local scale, are ineffective at minimizing the impacts of plastic pollution [28,29]. The presence of plastic pollution and subsequent interactions with marine fauna have been reported in various habitats of MPAs ranging from sessile coralligenous benthic habitats to pelagic habitats, including large mammal corridors such as the Pelagos Sanctuary [30,31].

Within the Balearic Islands, the MPA of Cabrera Marine-Terrestrial National Park (Cabrera MPA) is located off the south-eastern coast of Mallorca and is a hotspot for endemic marine and terrestrial species. Until now, few studies have quantified marine plastic within the park and its implications for marine species [32,33,34]. Seafloor sediment studies have highlighted concentrations of plastic fragments in shallow coastal sediments within Cabrera in addition to the presence of plastic additives such as benzyl butyl phthalate and Crelan^®^ within the seagrass meadows of *Posidonia oceanica* [32,33]. In the nearby MPA of the Menorca Channel, Ruiz-Orejón et al. (2019) [35], plastic particles floating on the sea surface range in size from 138,293 (±125,854) items/km^2^ in autumn and 347,783 (± 457,128) items/km^2^ in spring, while in the Cabrera MPA, Fagiano et al. (2022) [36] identified an abundance of plastic particles which were measured at 3.52 (±8.81) items/m^3^. With this in mind, there is still a lack of knowledge regarding the potential implications posed by marine plastic on marine diversity within Cabrera MPA.

Consequently, in this study we analyse plastic item interactions within the coastal ecosystem of Cabrera MPA with the following aims: (i) quantify the ingestion of APs across fish, sea urchins, sea cucumbers (echinoderms), bivalves (molluscs), and jellyfish (cnidarians); (ii) characterize the ingested items identified in the studied species; (iii) identify biotic and abiotic factors that might influence the ingestion of plastic items; and (iv) identify ingestion patterns based on taxonomic groups, trophic guilds, and habitats.

## 2. Materials and Methods

### 2.1. Study Area and Sample Collection

The ingestion of anthropogenic particles (APs, microplastics and other certified items of human origin such as fibres) were analysed in biota found within the coastal waters of Cabrera Marine-Terrestrial National Park (Cabrera MPA). The Cabrera MPA is the largest national park in Spain located off the southern coast of the Balearic Islands Archipelago in the western Mediterranean Sea, and the main currents include the Northern and Balearic currents to the north and the Algerian currents and gyres to the south (Balbin et al., 2014) [37]. It is currently managed by the Conselleria de Medi Ambient i Territori of the local government of the Balearic Islands. Currently, authorization is needed for the majority of activities including diving, sailing and anchoring while activities such as the extraction or collection of natural materials, hunting, and camping among others are strictly prohibited.

**Figure 1:**
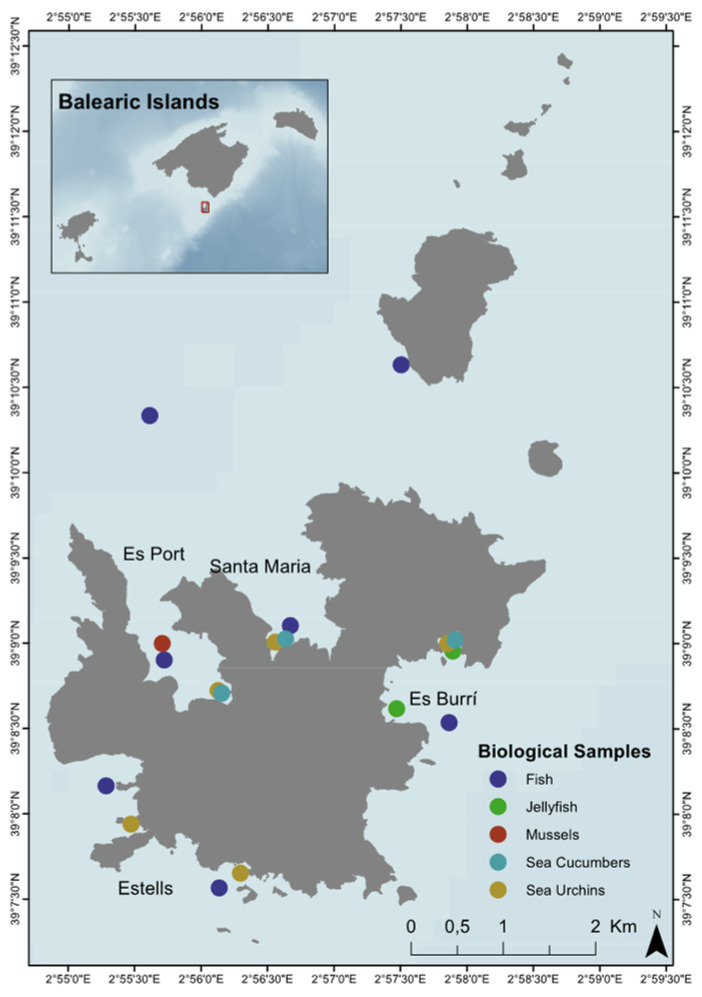
Study area and sampling sites for each of the species collected in the Cabrera National Park MPA located off the south-eastern coast of Mallorca in the Balearic Island Archipelago. The inset map indicates the location of the Cabrera National Park MPA with a red extent marker.

The field campaigns were performed during the 2019 and 2020 seasons within the Plastic Busters MPAs Interreg Med-project aiming to contribute to the maintenance of biodiversity and preservation of natural ecosystems in pelagic and coastal MPAs from marine litter pollution. Samples of fish, echinoderms, molluscs, and cnidarians were collected from several locations with various degrees of protection throughout the MPA (Figure 1). Located in the harbour area of Cabrera MPA, Es Port represents the most heavily impacted area by human activities as daytime and night-time anchoring and navigation are permitted at established buoys as well as transit of visitors (there is a small canteen and several beaches with public access). Regarding the Santa Maria, Enciola and Estells locations, these are strict no-take areas and public access is restricted. Finally, to the west of Cabrera MPA, Es Burrí is located in an area with moderate restrictions where daytime anchoring is permitted at a limited number of anchored buoys on the southernmost tip of the bay while navigation and anchoring are prohibited at the northern areas of this small bay. For the assessment of AP ingestion in the biota of the study area, a total of 313 individuals belonging to 17 different fish species and 8 invertebrate species (two species of sea urchins, three species of sea cucumbers, two species of molluscs and one cnidaria species) representative of pelagic, demersal benthic, and benthopelagic habitats were selected within the MPA.

### 2.2. Biota Samples

#### 2.2.1. Collection, Laboratory Processing and Biological Indices

The multispecies assessment was conducted within the marine protected area of the Cabrera Maritime-Terrestrial National Park (hereinafter Cabrera MPA) between 2019 and 2020 at various sites throughout the main island. The majority of the fish samples were collected in situ during the field work campaigns established within the Plastic Busters MPA consortium [38]. Three species of fish, *E. marginatus*, *T. alalunga,* and *M. surmuletus,* were provided by local professional fishermen fished in Cabrera MPA. The two molluscs *A. noae* and *M. galloprovincialis* were collected within the Es Port area of the harbour from submerged buoys at depths ranging from 5 to 12 m. The echinoderms were collected during scuba-diving surveys from the coastal sea bottoms, while the jellyfish were obtained during manta trawl sampling from the sea surface using a hand net.

All samples were processed in situ at the field laboratory in Cabrera MPA. For the fish species, the following biological parameters were recorded for all fish: total length (TL) (cm), weight (g), and gastrointestinal tract weight (g). The entire gastrointestinal tract (GIT) was extracted for each individual and stored at −20 °C inside a sterile, plastic zip-lock bag for post-AP analysis in the laboratory of the Oceanographic Centre of the Balearic Islands (IEO-CSIC) [39]. For each species of fish, the Fulton Condition Factor (K) and the Stomach Fullness Index were calculated as follows:

1. Fulton’s condition factor (K) = (total weight (g)/(total length (cm)^3^) × 100
2. Stomach Fullness Index (FI) = stomach content weight (g)/eviscerated weight (g) × 100

For the sea urchin and sea cucumber species, the disc width (cm) and the disc height (cm) of the body were measured with a calliper and weighed (g). For all species, the entire GIT was extracted and stored at −20 °C until further processing in the laboratory. For sea cucumbers, the square root of the length–width product index (SLW) [40] and for sea urchins, the Condition Index (CI) [41] were used to calculate the growth function applying the following formulas:

3. Square root of the length–width product index (SLW) = square root of length (cm) × width (cm)
4. Condition index (CI) = stomach content weight (g)/total weight (g) × 100

For the mollusc species, the total length (TL) (cm), width (cm), weight (g) soft tissue (g) and intestinal weight (g) were measured and the soft tissue was stored immediately at −20 °C for the posterior analysis of the AP. Additionally, the Mussels’ Condition Index (MCI) of each individual was determined as a ratio of soft tissue wet weight (g) to shell weight [42]:

5. Mussels condition Index (MCI) = wet weight of the soft tissue (g)/shell weight (g)

In terms of the jellyfish species *P. noctiluca*, at the two sites sampled for jellyfish, samples were collected with a manual net and kept in seawater until arrival at the field laboratory. Biological parameters for total length (cm), width (cm) and height (g) of the umbrella and tentacle length (cm) were measured.

Once in the laboratory, for each individual of the fish and echinoderm species, the GITs were removed from storage at −20 °C and defrosted at room temperature for subsequent chemical digestion process in 10% KOH for a period of 48–96 h in a bath at 50 °C. For the molluscs, whole organisms were digested following the same procedure of chemical digestion. The supernatant for each sample was filtered using a vacuum pump within a fume hood and filtered through a fibre glass filter (FILTER-LAB, pore size 20.0 µm, diameter 47 mm). Any remaining material was visually sorted under a stereomicroscope to ensure that no remaining APs were retained on the surface. The sea urchin and sea cucumber samples with large amounts of undigested organic matter (remnants of *Posidonia oceanica* leaves), were treated with 96° of ethanol to increase floatability of potential plastic/APs particles and isolate them from the organic matter [43]. For the species *P. noctiluca*, each individual was placed in a glass Petri dish and large visible items were separated from the individual and a second examination was performed under the stereomicroscope to remove any APs that may have adhered to the cavities. Additionally, each individual was rinsed with distilled water and the remaining water was examined under the stereomicroscope for possible APs.

Finally, independent of the isolation method according to the study species, all filters and visually sorted items were separated and characterized under a stereomicroscope (Euromex NZ 1903-S) with an optical enhancement of 6.7× to 40.5×.

#### 2.2.2. Characterization of Ingested Microparticles

All items found in each individual were characterized following the Plastic Busters MPAs Toolkit for marine biota [38]. All elements were classified by colour (white/transparent, white/opaque, red, green, blue, black, and others) and classified by fibres, fragments, films, and others. No items from the categories rope and filament, microbeads, and pellets were identified. For measurements, images of APs were taken with a CMEX 3.0 MP camera attached to the microscope and measured using special calibration software, ImageFocus^®^ 4.0. (Euromex software).

For polymer characterization, 285 items were determined using Fourier transform infrared (FTIR) spectroscopy (Bruker) in ATR (attenuated total reflection) mode. Items measuring > 300 µm were collected from the microscope slide and placed separately on the ATR unit to be analysed with the platinum ATR of the Tensor 27 spectrometer (Bruker, Germany). Considering that there were several very small items measuring <300 µm, these items were measured directly on the filters, using the ATR crystal unit attached to a microscope (micro-FTIR, single-element LN-MCT). A wavenumber range of 400–4000 cm^−1^ was used for the measurements and 8 scans per element were performed. Each spectrum was compared with spectra from a customized polymer library integrating different databases [44,45] and an in-house library generated with virgin and marinate reference polymers including various natural and synthetic materials following Fagiano et al. (2022) [34] using OPUS 6.5 software. Only samples with a hit quality index >700 (max. 1000) were accepted as confirmed polymers.

### 2.3. Data Analysis

To determine which biotic and abiotic factors contribute to AP ingestion in fish, echinoderms, molluscs, and cnidarians within the Cabrera MPA, each group of species was individually analysed. For the factors family, trophic guild and habitat, normal distribution was assessed using the Shapiro–Wilcox test and the data were found to be non-normally distributed; therefore, the Kruskal–Wallis (KW) test was applied to identify differences between the mean number of ingested APs and each factor for each species. For factors with more than two levels (e.g., location and fish species), a KW test was used to identify differences between the mean number of ingested items and each factor, while the Mann–Whitney U test (MW) was used for those factors with only two levels (e.g., year and mussel species).

For fish species, a total of 174 fish samples were collected between 2019 and 2020; however, for statistical purposes, only those species with a minimum of 5 individuals per sampled species were included in the statistical analyses. For fish, a KW was used to determine differences between locations and species while differences between years were analysed via an MW. A Spearman’s Rank Correlation was performed to determine significant correlations between the number of items ingested and FI and K for all species of fish. For echinoderms, an MW was used to determine differences between sampling year and a KW for differences between species and sampling location while Spearman’s Rank Correlation was performed to determine significant correlations between the number of ingested items and CI and SLW. For molluscs, MW tests were performed to assess differences according to sampling year and between species, while Spearman’s Rank Correlation was performed to determine significant correlations between the number of items ingested and the continuous biological coefficients for the MCI. Finally, for the cnidarians, an MW test was performed to determine differences between locations while Spearman’s Rank Correlation was performed to determine whether there were significant relationships between the number of ingested items and the total length and umbrella width of each individual. Dunn post hoc analyses for multiple comparisons were performed to evaluate statistical differences between groups for all KW and MW models with significant factors.

To identify an ecosystem approach that evaluates the overall ingestion of AP within the studied species of Cabrera MPA, an alluvial diagram of the family, trophic guilds (functional groups), habitat, and predominantly ingested items was assigned to each species considering the percentage of AP occurrence. This gives an indication of the predominant items of ingested items found in combination with their trophic guilds. Trophic guilds were assigned to each species following Macieira et al. (2021) and Pinheiro et al. (2018) [46,47] as: Herbivore (HERB)—species that consume a rich mass of macroalgae, turf algae, and detritus; Macro-carnivore (MCAR)—species that consume large mobile organisms, including invertebrates and fishes; Mobile Invertebrates Feeders (MIF)—fish that feed primarily on small benthic mobile invertebrates; Omnivores (OMN)—species that consume a variety of organisms, including both animals and plants; and Sessile Invertebrates Feeder (SIF). The habitats were assigned as benthic, pelagic, benthopelagic, and demersal. The percentage of occurrence indicates the number of individuals with APs in their gastrointestinal tracts. All data analyses were performed in RStudio version 3.6.4.

### 2.4. Quality Assurance and Control

During all laboratory work, to minimize contamination, all windows and doors remained closed, and technicians wore 100% cotton white laboratory coats. Glass and metal materials were used during all dissection processes and materials were thoroughly cleaned with filtered distilled water and 70% ethanol before and between manipulating each sample. During sample digestion, a procedural blank was run after every three samples, and one procedural blank was run for each sample during visual sorting. If items were found in the blanks, sample correction was performed by subtracting the number of items found in the blanks dependent on shape and colour to consider any possible background contamination during laboratory procedures. Overall, for fish an average of 0.24 items/ind. were found in the blanks, for invertebrates an average of 0.51 items/ind. and finally for molluscs an average of 1.7 items/ind. were identified.

## 3. Results

### 3.1. Biological Parameters

Overall, 313 individuals from 25 species obtained from the National Park of Cabrera MPA were analysed. A total of 174 fish of 17 different species were sampled and the biological parameters for total length (TL, cm), weight (g), gastrointestinal weight (g), FI and K for the fish are given in Table 1. The largest species sampled were the most predatory species, *T. alalunga* (TL, 64.9 ± 2.75 cm) (mean ± standard deviation) and *E. marginatus* (TL, 56.7 ± 4.89 cm) while the smallest species sampled were *A. anthias* (TL, 13.6 ± NA cm) and *C. julis* (TL, 14.75 ± 2.05 cm) (Table 1). For echinoderms, the largest sea cucumbers belonged to the *H. tubulosa* species (TL, 27.8 ± 4.15 cm) while *H. poli* (TL, 16.13 ± 4.15 cm) and *H. forskalii* (TL, 16.02 ± 2.66 cm) were very similar in size (Table 1). Regarding the two filter feeding molluscs analysed, *A. noae* was overall slightly larger (length, 6.11 ± 0.59 cm) compared to *M. galloprovincialis* (length, 5.37 ± 1.80 cm). Finally, the *Pelagia noctiluca* cnidaria species *(n = 31)* analysed showed a total length (TL) of 10.32 ± 2.68 cm (Table 1).

### 3.2. Ingestion and Characterization

Anthropogenic particles (APs) were found in 16 GIT of the 17 fish species samples and a total of 554 items were identified. In general, the occurrence of APs ranged from 0 to 100% regarding the species and with an average of ingested items ranging from zero ingestion in *T. trachurus* to 14.5 ± 7.8 items/ind. in *B. boops* (Table 2). With regard to the types of items ingested, fibres were the most common items found (75%), present in all species except *C. auratus, S. scombrus,* and *T. trachurus*. Concerning species that had ingested fibres, the occurrence of fibres ranged from 16% (*S. cantharus*) to 100% (*C. julis*) of the total identified items. The next most common type of AP items in fish were fragments (18%) and four species contained fragments in their gastrointestinal tracts and the incidence ranged from 4% (*S. cantharus*) to 100% (*C. auratus*). In terms of films, only five species had ingested films of which two species almost exclusively ingested films, *S. cantharus* (80%) and *S. scombrus* (100%). Regarding the ingested particles, since the pore size of the filters was 20 µm, this was our limit of detection for the minimum size of APs, although the smallest items observed measured 33 µm. Within the GITs of fish, the average size ranged from 0.07 ± NA mm in items ingested by *A. anthias* to 2.57 ± 0.89 mm in items ingested by *S. scombrus*. *S. cantharus* was the only fish species to ingest items larger than 5 mm with an average size of 5.56 ± 4.18 mm (Table 2). For ingested APs, the sizes ranged from 33 μm to 1562.1 μm (fish—minimum to maximum), from 115.4 μm to 5170.23 μm (molluscs), and from 141.1 μm to 9997.8 μm (invertebrates).

For echinoderms, the mean ingestion of sea cucumbers ranged from 8.6 ± 8.6 items/ind. (*H. poli*) to 16.0 ± 7.8 items/ind. (*H. tubulosa*) and both species had a 100% incidence of ingestion, while *H. poli* had a 64% incidence (Table 2). Fibres were the dominant type of AP for all species (71.4%), followed by other types of items (14%) and fragments (12.5%). In addition to these items, *H. poli* had ingested other items of AP such as glass particles, carbon, ceramics, and rusted metal fragments. For sea urchins, similar items were ingested, although the occurrence and overall average number of items/ind. were much lower (*A. lixula*, 1.1 ± 2.4 items/ind.; *P. lividus,* 1.6 ± 3.3 items/ind.).

With regard to the molluscs, the bivalve *A. noae* showed an average of 5.0 ± 5.5 items/ind. within the whole organisms and an overall occurrence of 72%, while *M. galloprovincialis* exhibited 4.2 ± 3.2 items/ind. and an overall occurrence of 96% (Table 2). Similar amounts of AP were found in both species, with fibres representing the main items (79.8–80%) followed by fragments (17–18%). Furthermore, for cnidarians, an overall occurrence of 35.5% was identified and most items found within the umbrellas of the jellyfish species *P. noctiluca* were mostly fragments (56.5%) (Table 2).

**Figure 2:**
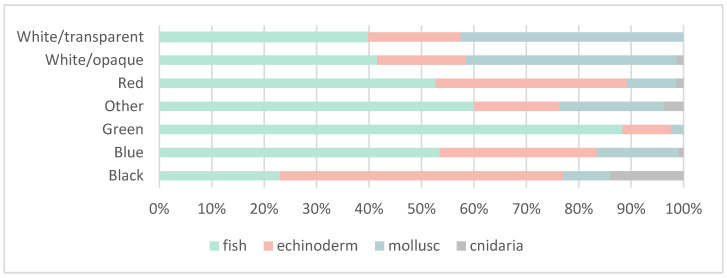
Bar graph regarding the colour of identified items from the gastrointestinal contents of fish (green), echinoderm (red), mollusc (blue) and cnidaria (grey). Each bar indicates the percentage of occurrence of each colour group according to analysed species.

In terms of colour, the most commonly identified colour was blue (46.5%) followed by white/transparent (22.9%). For the fish species, most of the items ingested were blue (51.4%) followed by white/transparent (19%), similar to the overall results and the results with respect to the mollusc species (white/transparent 43.9%; blue 32.8%) (Figure 2). For the echinoderms, the dominant colours were blue (51.0%) and black (17.3%) while in jellyfish, the dominant colour was also black 61% followed by blue 22%.

#### Polymer Characterization

**Figure 3:**
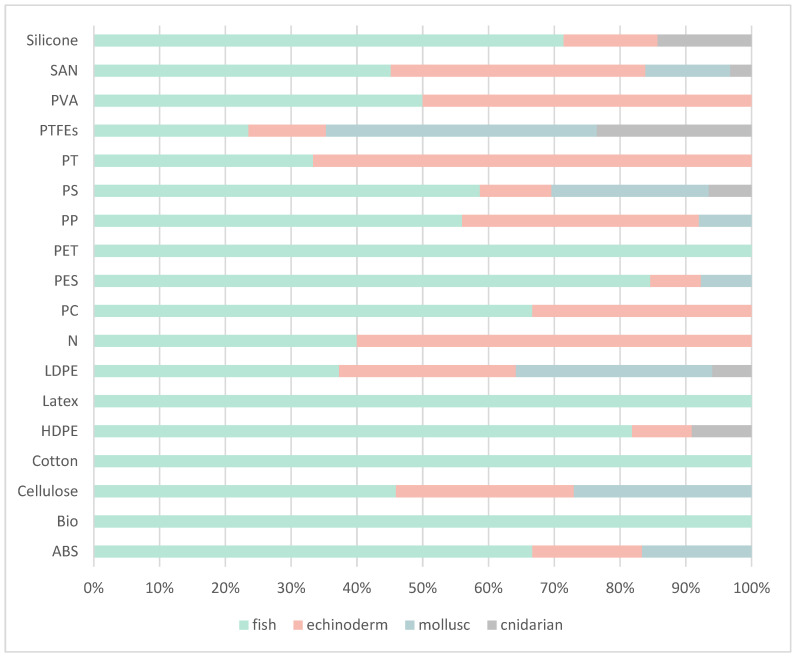
Bar graph of the polymers identified via ATR-FITR and micro-FTIR according to chemical composition: acrylonitrile butadiene styrene (ABS), biodegradable plastics (Bio), Cellulose, cotton, low-density polyethylene (LDPE), high-density polyethylene (HDPE), latex, nylon (N), polycarbonate (PC), polytetrafluoroethylene (PTFE), polyamide (PA), polyester (PES), polyethylene terephthalate (PET), polypropylene (PP), polystyrene (PS), styrene–butadiene rubber (SBR), paint (PT), poly (vinyl alcohol) (PVA), polyvinylidene fluoride (PVDF) and styrene–acrylonitrile (SAN).

A total of 299 AP items (fragments, films, fibres, and filaments) were analysed with ATR-FTIR and micro-FTIR, of which 53% of the items were from fish, 23% from echinoderms, 19% from molluscs, and 5% from jellyfish. Sample spectra can be found in Figure S1. According to the results, there was large variability in polymer type of both artificial and natural origin ingested by the species, with 18 different types of polymers identified. For all species, the most common polymers identified were low-density polyethylene (LDPE) (22%) and polystyrene (PS, 15%). Styrene–acrylonitrile (SAN; 7–17%), polytetrafluoroethylene (PTFE; 3–29%), PS (7–21%), and LDPE (15–29%) were polymers found in all species with varying percentages of occurrence (Figure 3). Fish species ingested the widest variety of anthropogenic items as all polymers were identified (Figure 3). The two species of molluscs analysed, ingested eight types of polymers including SAN, PTFE, PS, PES, Cellulose, ABS, PP and LDPE (Figure 3). For jellyfish, the most common polymers comprising the APs ingested were LDPE (29%) and PTFE (29%). Finally, there were two biodegradable polymers (4%) found in fish GITs, one being Ecoflex^®^, a biodegradable and compostable polymer and the other being polycaprolactone, a biodegradable polyester.

### 3.3. Data Analyses

**Figure 4:**
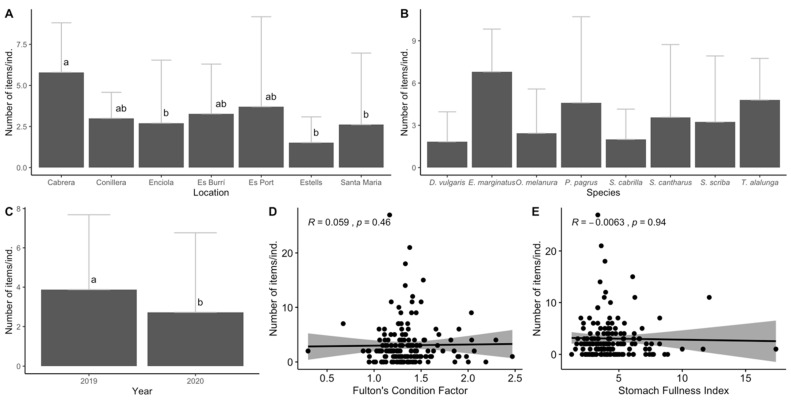
Results for the ingestion of anthropogenic particles (AP) in fish samples according to location (**A**), species (**B**) and year (**C**), as well as the Spearman rank correlation between Fulton’s condition factor (**D**) and the stomach fullness index (**E**). The error bars for (**A**–**C**) indicate a 95% confidence interval, and significant differences are indicated by different letters. The significance level was established at *p* < 0.05.

In general, fish within Cabrera MPA ingested an average of 3.16 ± 4.16 items/ind. In terms of location, species samples collected by professional fishermen in Cabrera waters (*E. marginatus* and *T. alalunga*), showed significantly higher ingestion values than ingestion values of species sampled within the no-take areas of Enciola, Estells and Santa Maria (KW, *p* < 0.01; Figure 4A). Regarding the species, although the ingestion values ranged from 1.75 ± 2.18 items/ind. in *D. vulgaris* to 6.8 ± 3.04 items/ind. in *E. marginatus*, no significant differences were identified between the sample species (KW, *p* > 0.05; Figure 4B). In terms of differences between years, significant differences were observed with higher amounts of ingested APs in the sampled species collected in 2019 compared to 2020 (KW, *p* < 0.05; Figure 4C). For the overall fitness of all species, no correlation was identified between the number of items ingested and either the Fulton’s Condition Factor (R = 0.059, *p* > 0.05; Figure 4D) or the Stomach Fullness Index (R = −0.006, *p* > 0.05; Figure 4E).

**Figure 5:**
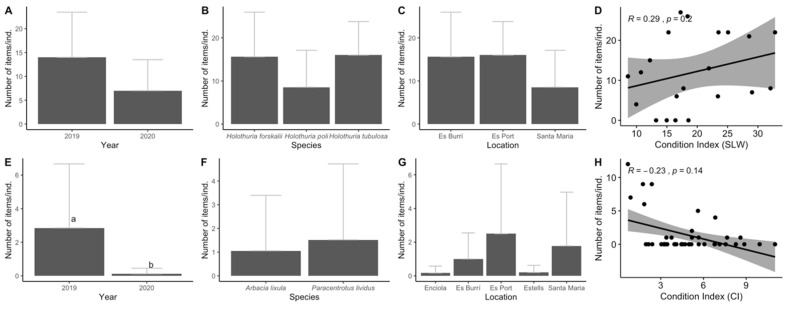
Results for the ingestion of anthropogenic particles (APs) in echinoderm samples for sea cucumbers (Holothuria sp.): year (**A**), species (**B**), location (**C**) and square root of the length–width index (SLW) (**D**) and for sea urchins: year (**E**), species (**F**), and location (**G**) as well as the Spearman’s rank correlation (significance level at *p* = 0.05) between Condition Index (CI) (**H**). The error bars indicate a 95% confidence interval, and significant differences are indicated by different letters. The significance level was established at *p* < 0.05.

Low variability in APs ingested in terms of species, location, and condition index was observed among echinoderms. For sea cucumbers, no significant differences were found between years, locations and species regarding AP ingestion (Figure 5A–C) and in terms of the square root of the length–width index (SLW), no correlation was found between SLW and the number of items ingested (R = −0.05, *p* > 0.05; Figure 5H). For sea urchins, significant differences were found between years (MW, *p* < 0.001; Figure 5E) with almost twice as many ingested items in individuals from 2019 compared to those from 2020. No significant differences were observed between species nor between locations despite the fact that the individuals sampled from Es Port and within Santa Maria ingested almost threefold the average number of items compared to those sampled in Enciola and Estells (Figure 5F,G). Additionally, no significant correlation was identified between the number of ingested items and the condition index (R = −0.23, *p* > 0.05; Figure 5H).

**Figure 6:**
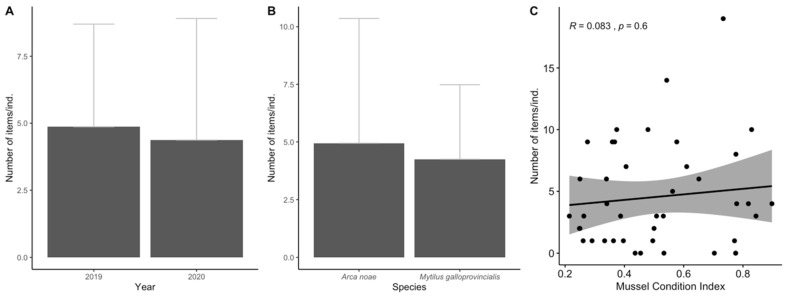
Results for the ingestion of anthropogenic particles (APs) in mollusc samples for: year (**A**), species (**B**) as well as the Spearman’s rank correlation (significance level at *p* < 0.05) between Mussel Condition Index and number of APs ingestion (**C**). Error bars for indicate a 95% confidence interval and significant differences are indicated by different letters. The significance level was established at *p* < 0.05.

Regarding the mollusc species, no significant differences were found between the AP ingestion values according to years (MW, *p* > 0.05) nor between the species, although *A. noae* had slightly higher average ingestion values compared to *M. galloprovincialis* (MW, *p* > 0.05; Figure 6A,B). Additionally, no significant correlation between the mussel condition index and the number of ingested items/ind. was observed (R = 0.08, *p* > 0.05; Figure 6C).

**Figure 7:**
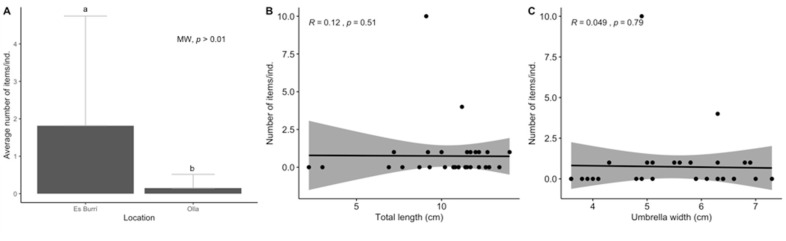
Results for the ingestion of anthropogenic particles in cnidaria samples: location (**A**) and Spearman rank correlation between the total length and the number of APs ingestion (significance level at *p* = 0.05) (**B**) and the width of the umbrella and the number of APs ingestion (**C**). The error bars for location indicate a 95% confidence interval and the significant differences are indicated by different letters. The significance level was established at *p* < 0.05.

The *P. noctiluca* jellyfish samples showed micro-scale variability in plastic ingestion with some individuals exhibiting a twofold increase in concentration of plastic items (MW, *p* < 0.01; Figure 7A). In terms of overall total length and width of the umbrella, no significant correlation was observed between the biological parameters and the number of ingested items (Figure 7B,C).

**Figure 8:**
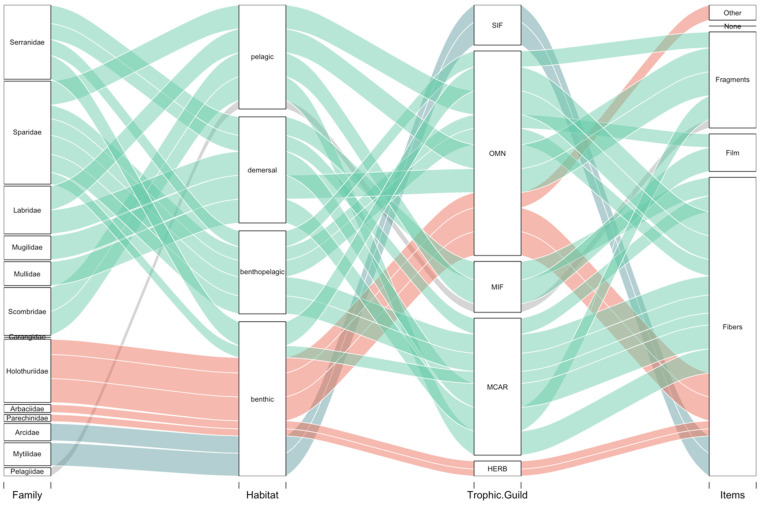
Alluvial diagram of the species by family: fish (green), echinoderms (red), bivalves (blue), cnidaria (grey); habitat: pelagic, benthopelagic, demersal, and benthic; trophic guild: mobile invertebrate feeders (MIF), macro-carnivores (MCAR), omnivores (OMN), sessile invertebrate feeder (SIF) and ingested items: fragments, fibres, films and other. The width of the flows considers the percent occurrence of ingestion for each of the species.

An exploratory data analysis on family, trophic guilds, habitats, and predominant items for each species highlights that most fish species are pelagic, demersal, or benthopelagic, primarily ingesting fibres and fragments (Figure 8, Table 2). For invertebrates, sea cucumbers (omnivores) and sea urchins (herbivores), most species had an ingestion occurrence larger than 75% (*H. forskalii* and *H. tubulosa*), except for the sea cucumber species *H. poli* with an ingestion occurrence of >60%. On the other hand, molluscs are exclusively sessile invertebrate feeders (SIF) and showed an occurrence between 50–100% dependent on the species. Finally, individuals from Pelagidae species were mobile invertebrate feeders (MIF) and had a low ingestion occurrence of 35.5%.

**Figure 9:**
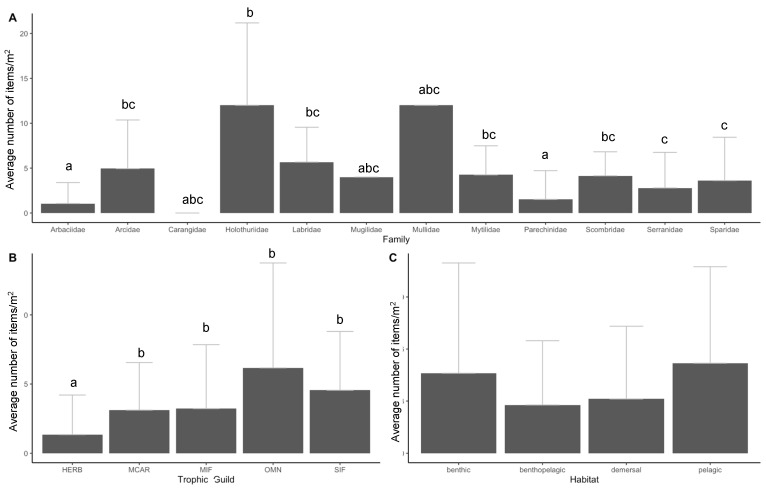
The mean number of anthropogenic particles (mean ± standard deviation) found in the study species regarding family (**A**), trophic guild (**B**) and habitat (**C**). Error bars indicate a 95% confidence interval and significant differences are indicated by different letters. The significance level was established at *p* < 0.05.

In terms of contributing factors to ingestion, significant differences were found between the mean number of items ingested and the families (KW, *p* < 0.001; Figure 9A). Multiple comparisons indicate that individuals from the family Arbaciidae and Parenchinidae were significantly different from Arcidae, Holothuriidae, Labridae, Scombridae, Serranidae, and Sparidae (Figure 9A). Significant differences between the mean number of ingested items were also found between trophic guilds (KW, *p* < 0.001). A multiple comparison Dunn’s test indicated that herbivores ingested significantly less amounts of APs with respect to the other four trophic guilds (MCAR, MIF, OMN, and SIF) while no significant differences were found among the four remaining trophic guilds (MCAR, MIF, OMN, and SIF) (Figure 9B). In terms of habitats, no significant differences were found between the mean number of ingested items and habitats (KW, *p* > 0.05; Figure 9C).

## 4. Discussion

The increased reporting of anthropogenic particles in the marine environment raises alarms concerning the impact on marine diversity, not only in coastal regions near coastal cities where marine litter is primarily sourced, but also in remote areas and marine protected areas [27,48]. Considering that the Mediterranean Sea is a hotspot for biodiversity and coastal areas are at the highest risk regarding human activity and pressure, it is essential to quantify this impact using harmonized protocols and establish bioindicator species for monitoring between regions. Previous risk assessments conducted for the Mediterranean Sea have identified marine diversity across several taxonomic classes and habitats in coastal areas exposed to plastic litter and the associated effects, especially in MPAs and coastal areas [9,28]. In this study, ingestion was present in 96% of the species analysed from all locations and varied between species, families, and trophic guilds.

Most of the fish species (99%) had ingested APs and for those species with more than five individuals sampled, no significant differences were identified between species and ingestion. Despite this, the differences between locations were significant, indicating heterogeneous availability of APs on a small scale within the MPA. Nonetheless, there was a large amount of variability in ingestion according to species. For example, *P. pagrus* had a mean number of 4.6 ± 6.1 items/ind. ingested with an 80% occurrence, which is much higher than previous reported ingestion rates with a mean of 1.44 items and a 56% ingestion rate [49]. For *S. cantharus*, 57% of the individuals ingested AP with a mean of 3.6 ± 6.1 items/ind., which is in agreement with the individuals sampled off the Italian coast near the Costa Concordia shipwreck, where the number of particles in positive stomachs ranged from 2.3 ± 1.15 to 5 ± 0. Concerning the bioindicator species, although a low number of *B. boops* individuals were sampled (n = 2), this species exhibited the highest ingestion rate (14.5 ± 7.8 items/ind.) followed by *M. surmuletus* (12 APs) (n = 2). These values are much higher than those reported in previous studies where *B. boops* samples were found to have an average of 2.7 items/ind. [50] and 0.5–1.68 items/ind. [51], while in the Balearic Sea, Nadal et al. (2016) 10 found an average of 3.75 items/ind. and Rios-Fuster et al. (2019) 14 found a lower AP ingestion average of 0.33 ± 0.87 items/ind. Considering the elevated number of APs found in fish GITs, monitoring of fish species within the MPA of Cabrera should be continued.

For the sea cucumbers, most of the items ingested were fibres (42–87%). The high frequency of ingested fibres is in agreement with the findings of Sayogo et al., 2020 [52], where the sea cucumber *Apostichopus japonicus* from several locations in China exhibited fibres as the main ingested item. The high concentrations of fibres located on the seafloor may be attributed to their low density, and thus the fibres are easily transported downwards to the seafloor by bottom currents as stated in deep sea areas [53,54]. An interesting result was the presence of carbon and glass particles inside sea cucumber samples. Previous studies of sediment samples in Cabrera MPA identified up to 0.90 ± 0.10 items/g [33] and scuba-diving surveys of the region highlight the high concentration of plastic and glass material on the seafloor [55]. These areas are increasingly becoming sinks for marine litter, which can lead to negative impacts on sea cucumber health in Cabrera MPA.

With regard to sea urchins, in the study area the overall ingestion occurrence was 33% for *A. lixula* (average 1.1 ± 2.4 items/ind.) and 30 % for *P. lividus* (average of 1.6 ± 3.3 items/ind.). Similar ingestion values were observed in sea urchins off the coast of northern China (2.20 ± 1.50 to 10.04 ± 8.46 items/ind.) [56]. These values are lower than those previously observed in the eastern Aegean Sea, where Hennicke et al. (2021) [57] observed an average of 28.95 items/ind. In laboratory studies, *P. lividus* was found to have a high capacity to fragment marine plastics with the potential to create 91.7 plastic fragments over a 10-day period [58]. This indicates that sea urchins can break marine plastics into smaller particles and could be a potential source of plastic fragmentation and downsizing marine litter increasing the ability for bioaccumulation processes to occur. Additional laboratory studies in sea urchins have identified negative health effects in individuals fed with polystyrene beads, where a stress-related impact on circulating immune cells was identified [59] and *Pseudechinus huttoni* larva exposed to microplastics resulted in oxidative damage in lipids and proteins [60]. Studies conducted on wild individual species and in laboratory experiments indicate that sea urchins can be harmed by APs in marine environments, in addition to increasing the fragmentation of plastics.

Molluscs, specifically mussels, have been globally identified as bioindicator species for monitoring plastic pollution [61] by being sessile species and providing essential information on plastic pollution and pollutant concentrations [62]. In this study, two species of molluscs *M. galloprovincialis* and *A. noae* were analysed and ingestion was evident in both species. *M. galloprovincialis* showed a mean ingestion value of 1.10 ± 0.97, in agreement with studies performed in the Northern Ionian Sea exhibiting 47.2% occurrence and mean value of 0.8 ± 0.2 [63]. Ingestion of APs in aquaculture experiments highlighted that the species ingested microplastics ranging from 4.54 ± 0.34 to 18.2 ± 4.16 items/ind. [23]. In terms of the ingestion in sea cucumbers, sea urchins and molluscs, another bioindicator species not included in this study could be other benthic sessile species such as sea anemones. For example, Morais et al. (2020) [64] observed the ingestion of meso- (5.01–25 mm) and microplastics (1 µm–5 1 mm) by the sea anemone *Bunodosoma cangicum* and a weak correlation between the weight of the anemones and the number of plastic particles. These findings are a cause for concern as laboratory studies have indicated chemicals associated with plastic pellets can trigger feeding responses in sea anemones [65]. Additionally, the potentially toxicological effects of long-term exposure in marine and aquaculture settings have been shown to cause an antioxidant response in mussels, inducing several effects at the transcriptional and cellular levels, highlighting potential risks to the health condition of organisms [23,66]. To our knowledge, this is the first study to identify the occurrence of APs in *A. noae*. Although previous studies have identified the accumulation of trace elements in this species [67], we propose that this species also be considered a bioindicator species as an indirect method of measuring AP pollution in the marine environment.

This study highlights the entrapment of plastic litter within the umbrella of the jellyfish species *P. noctiluca* in 35% of the individuals in Cabrera MPA. In a similar study from the Canary Islands, plastic litter items either within jellyfish umbrella or tentacles accounted for 97% of the jellyfish analysed [68]. However, in this study no plastic particles were encountered in the tentacles of jellyfish at Cabrera MPA. Retention time of plastic litter in different species is highly variable and species-dependent, representing a knowledge gap in species behaviour and feeding selectivity. In general, considering both the abundance of *P. noctiluca* in the Mediterranean Sea Basin and the results of this study, jellyfish could be proposed as a candidate bioindicator species for plastic pollution.

Regarding the type of polymers ingested, the most common type was LDPE (22.1%) followed by PS (15.1%) and SAN (10.5%). In addition, several items were identified as persistent organic pollutants (POPs), primarily polytetrafluoroethylene (PTFEs). In invertebrates, specifically the mussel species, PTFE was one of the most common items found (12.5%) and was ingested by sea urchins and fish. Dahl et al. (2021) [32] identified PTFE in sediments samples within the same region in Cabrera MPA. Although studies on the environmental consequences of PCFs are scarce, Liu et al. (2014) [69] highlighted that the exposure of green mussels *Perna viridis* to this type of molecule can induce a series of adverse effects at different biological levels, including oxidative stress, DNA damage, membrane instability, among others.

In terms of trophic guilds, herbivores tended to ingest significantly fewer APs compared to the other categories of trophic guilds. In this study, the two sea urchin species were the only primarily herbivore species and similar results were evident in other studies where sea urchins ingested considerably fewer APs compared to other species [56]. Future monitoring may include herbivorous fish species such as *Sarpa salpa*. A recent study by Abidli et al., 2021 [70] identified very high ingestion rates with an average of 42.00 ± 6.08 items individual^−1^. Considering the other trophic guilds in this study (MIF, SIF, OMN, and MCAR), our results align with those reported by Dantas et al. (2020) [15], where no differences were found in fish species with zoobenthivorous or opportunistic/omnivorous diets, an indication that plastic intake was not dependent on feeding habits. Future studies would benefit from including fish species that are exclusively herbivores, which may provide information on whether the presence of APs is also affecting this specific trophic guild.

Regarding the different habitats, species representative of benthic, demersal, benthopelagic and pelagic species were found to have no significant differences according to AP ingestion, indicating that the presence of APs throughout Cabrera MPA is independent of habitat type. Although no significant differences were found between habitats, pelagic species ingested slightly more APs items than species from other habitats. Similar results were also found in Avio et al. (2020) [71], where no significant differences were observed regarding plastic ingestion between species of different habitats, although organisms in benthopelagic habitats did present a higher frequency of ingestion. In terms of overall marine diversity, a risk assessment conducted on marine diversity within the Mediterranean Sea identified demersal species from various taxonomic classes to be at slightly higher risk of ingesting plastics than pelagic species [9]. Considering these results, a multispecies approach is recommended for biomonitoring strategies in accordance with the research work by Avio et al. (2020) [71].

## 5. Conclusions

Overall, the results from this study highlight the ingestion of anthropogenic particles of varying chemical properties identified within the GITs of 25 different species at an MPA in the western Mediterranean Sea. Both the presence and ingestion of these particles were evident and the multispecies approach provided an ecological assessment of the impact and distribution of anthropogenic particles in the food web by considering species from different habitats and trophic guilds throughout the MPA of Cabrera. The results of this study provide a baseline for comparability among other regions and other MPAs in the Mediterranean Sea and highlight the importance of continued biomonitoring using multi-species assessment.

## Figures and Tables

**Table 1:** Summary of biometric parameters for all species surveyed during the 2019 and 2020 scientific surveys in Cabrera MPA, according to: group of species (fish, echinoderma, mollusc and cnidaria), family, species, number of individuals (N), total length (cm), weight (g), gastrointestinal tract (GIT) weight (g), Fullness Index, Fulton’s Condition Factor, disc width (cm), height (cm), weight (g), soft tissue (g), shells (g), height (g), and tentacle length (cm). For those species with one individual, the standard deviation was not available (NA).

Type	Family	Species	Number of Individuals	Total Length (cm)	Weight (g)	GIT Weight (g)	FI	FCI
Fish	Serranidae	*Anthias anthias*	1	13.6 ± NA	24.8 ± NA	0.50 ± NA	2.0 ± NA	0.99 ± NA
	Sparidae	*Boops boops*	2	25.75 ± 1.62	142.8 ± 43.85	14.55 ± 3.60	11.2 ± 1.09	0.82 ± 0.10
	Mugilidae	*Chelon auratus*	1	37.0 ± NA	427.20 ± NA	50.0 ± NA	4.45 ± NA	0.99 ± NA
	Labridae	*Coris julis*	2	14.75 ± 2.05	32.75 ± 12.5	1.1 ± 0.14	7.86 ± 2.93	1.83 ± 0.02
	Sparidae	*Diplodus annularis*	4	15.3 ± 0.82	65.67 ± 9.28	4.43 ± 0.32	5.21 ± 1. 86	1.58 ± 0.04
	Sparidae	*Diplodus vulgaris*	12	21.75 ± 2.26	167.18 ± 52.58	7.83 ± 2.31	3.33 ± 1.35	1.40 ± 0.28
	Serranidae	*Epinephelus marginatus*	5	56.7 ± 4.89	2584 ± 572.65	77.52 ± 17.18	4.79 ± 0	1.24 ± 0.09
	Labridae	*Labrus merula*	1	24.8 ± NA	188.40 ± NA	8.40 ± NA	13.72 ± NA	0.84 ± NA
	Mullidae	*Mullus surmuletus*	1	29.0 ± NA	296 ± NA	15.5 ± NA	5.71 ± NA	1.21 ± NA
	Sparidae	*Oblada melanura*	16	22.56 ± 2.93	167.97 ± 66.43	6.9 ± 6.12	4.15 ± 2.68	1.40 ± 0.24
	Sparidae	*Pagrus pagrus*	5	17.82 ± 3.57	97.22 ± 63.61	6.16 ± 5.75	6.2 ± 1.60	1.59 ± 0.12
	Scombridae	*Scomber scombrus*	2	34.3 ± 1.83	394.5 ± 65.05	25.8 ± 1.70	7.74 ± 1.78	0.97 ± 0.01
	Serranidae	*Serranus cabrilla*	33	18.89 ± 4.91	87.1 ± 55.59	2.65 ± 1.95	3.28 ± 1.24	1.12 ± 0.17
	Serranidae	*Serranus scriba*	76	16.55 ± 3.12	64.82 ± 33.54	2.71 ± 3.38	4.79 ± 2.35	1.48 ± 1.55
	Sparidae	*Spondyliosoma cantharus*	7	21.58 ± 3.89	168.2 ± 70.60	7.1 ± 3.38	4.63 ± 0.98	1.56 ± 0.31
	Scombridae	*Thunnus alalunga*	5	64.9 ± 2.75	5866 ± 666.96	175.98 ± 20.01	3.33 ± 0	2.15 ± 0.23
	Carangidae	*Trachurus trachurus*	1	35.7 ± NA	311.9 ± NA	6.3 ± NA	2.49 ± NA	0.69 ± NA
			**N**	**Total Length (cm)**	**Disc Width (cm)**	**Height (cm)**	**Weight (g)**	**Gut Weight (g)**
Echinoderma	Holothuriidae	*Holothuria forskalii*	5	16.02 ± 2.66	4.88 ± 0.49	3.38 ± 0.73	168.50 ± 36.72	30.44 ± 4.67
	Holothuriidae	*Holothuria poli*	11	16.13 ± 4.15	3.68 ± 1.01	2.97 ± 0.72	138.28 ± 47.86	22.13 ± 15.86
	Holothuriidae	*Holothuria tubulosa*	5	27.8 ± 4.15	5.56 ± 0.73	4.06 ± 0.43	390.80 ± 21.85	66.22 ± 11.16
	Arbaciidae	*Arbacia lixula*	18	-	4.79 ± 0.83	2.45 ± 0.56	49.52 ± 16.72	2.09 ± 1.17
	Parechinidae	*Paracentrotus lividus*	27	-	5.18 ± 0.67	3.01 ± 0.58	61.93 ± 19.68	2.98 ± 1.46
			**N**	**Length (cm)**	**Width (cm)**	**Weight (g)**	**Soft Tissue (g)**	**Shells (g)**
Molluscs	Arcidae	*Arca noae*	18	6.11 ± 0.59	2.5 ± 0.29	24.9 ± 4.45	8.41 ± 2.11	12.89 ± 2.12
	Mytilidae	*Mytilus galloprovincialis*	24	5.37 ± 1.80	3.0 ± 0.98	22.01± 16.94	3.41 ± 2.34	9.04 ± 6.34
			**N**	**Length (cm)**	**Width (cm)**	**Height (cm)**	**Tentacle length (cm)**	
Cnidaria	Pelagiidae	*Pelagia noctiluca*	31	10.32 ± 2.68	5.52 ± 1.11	2.1 ± 0.60	8.78 ± 1.44	

**Table 2:** Summary of the mean and standard deviation (mean ± SD) values of ingested anthropogenic particles per individual (items/ind.), frequency of anthropogenic particles (APs) occurrence (%), mean and standard deviation (mean ± SD) values of size of identified items (mm) and percentage of occurrence (%) of items according to item shape (fibres, fragment, film and other) identified in the studied species.

Type	Species	Habitat	Items/ind	% Occurence	Size (mm)	Shape % Items/Ind.			
			(mean ± SD)		mean ± SD	Fibres	Fragment	Film	Other
Fish	*Anthias anthias*	benthic	6.0 ± NA	100.0	0.065 ± NA	83.3	16.7	0.0	0.0
	*Boops boops*	pelagic	14.5 ± 7.8	100.0	1.00 ± 1.99	55.2	34.5	10.3	0.0
	*Chelon auratus*	pelagic	5.0 ± NA	100.0	0.30 ± 0.05	0.0	100.0	0.0	0.0
	*Coris julis*	benthic	1.0 ± 1.4	50.0	0.34 ± 0.16	100.0	0.0	0.0	0.0
	*Diplodus annularis*	benthopelagic	3.3 ± 4.0	75.0	0.34 ± 0.16	61.5	38.5	0.0	0.0
	*Diplodus vulgaris*	benthopelagic	1.8 ± 2.2	66.7	1.34 ± 1.69	42.9	52.4	0.0	4.8
	*Epinephelus marginatus*	demersal	6.8 ± 3.0	100.0	1.75 ± 1.52	76.5	23.5	0.0	0.0
	*Labrus merula*	demersal	4.0 ± NA	100.0	0.60 ± 0.16	25.0	75.0	0.0	0.0
	*Mullus surmuletus*	demersal	12 ± NA	100.0	0.71 ± 0.20	25.0	75.0	0.0	0.0
	*Oblada melanura*	benthopelagic	2.4 ± 3.2	68.8	1.99 ± 1.92	74.4	25.6	0.0	0.0
	*Pagrus pagrus*	benthopelagic	4.6 ± 6.1	80.0	2.04 ± NA	91.3	8.7	0.0	0.0
	*Scomber scombrus*	pelagic	2.5 ± 0.7	100.0	2.57 ± 0.89	0.0	0.0	100.0	0.0
	*Serranus cabrilla*	demersal	2 ± 2.2	66.7	0.31 ± 0.18	86.4	13.6	0.0	0.0
	*Serranus scriba*	demersal	3.2 ± 4.7	77.6	1.31 ± 1.28	89.4	9.3	0.8	0.4
	*Spondyliosoma cantharus*	benthopelagic	3.6 ± 5.2	57.1	5.56 ± 4.18	16.0	4.0	80.0	0.0
	*Thunnus alalunga*	pelagic	4.8 ± 3.0	100.0	1.59 ± 0.88	66.7	12.5	20.8	0.0
	*Trachurus trachurus*	pelagic	0 ± NA	0.0	-	0.0	0.0	0.0	0.0
Echinoderms	*Holothuria forskalii*	benthic	15.6 ± 10.4	100.0	1.70 ± 1.87	88.5	9.0	0.0	2.6
	*Holothuria poli*	benthic	8.6 ± 8.6	63.6	2.10 ± 1.55	42.6	13.8	0.0	43.6
	*Holothuria tubulosa*	benthic	16 ± 7.8	100.0	1.63 ± 1.24	87.5	6.3	3.8	2.5
	*Arbacia lixula*	benthic	1.1 ± 2.4	33.3	3.80 ± 2.67	89.5	10.5	0.0	0.0
	*Paracentrotus lividus*	benthic	1.6 ± 3.3	29.6	1.87 ± 1.65	65.9	29.3	4.9	0.0
Molluscs	*Arca noae*	benthic	5 ± 5.5	72.2	0.63± 0.57	79.8	2.2	18.0	0.0
	*Mytilus galloprovincialis*	benthic	4.2 ± 3.3	95.8	1.10 ± 0.97	80.0	2.0	17.0	1.0
Cnidaria	*Pelagia noctiluca*	pelagic	4.6 ± 3.9	35.5	-	39.1	56.5	0.0	4.3

## Data Availability

Data are available upon request.

## Supplementary Materials

The following are available online at https://www.mdpi.com/article/10.3390/biology11101375/s1, Figure S1: Example spectra for the following: (A) blue fiber of the material cellulose acetate found in an invertebrate (FTIR), (B) blue fiber of the biodegradable material Ecoflex® found in a fish species (FTIR) and (C) black fragment of polyethylene terephthalate found in a fish species (ATR-FTIR). All items had a quality hit >70%.
