# Supplementary material for: Multispecies Assessment of Anthropogenic Particle Ingestion in a Marine Protected Area
Source: Biology (Basel). 2022 Sep 20;11(10):1375. doi: 10.3390/biology11101375 (PMC9598462; doi:10.3390/biology11101375)
Supplement: Supplementary file 1 [file biology-11-01375-s001.zip › biology-1866571-supplementary.pdf]

biology

MDPI

Supplementary Materials

Multispecies Assessment of Anthropogenic Particle Ingestion in a Marine Protected Area

Montserrat Compa, Carme Alomar, María Francesca López Cortès, Beatriz Rios-Fuster, Mercè Morató, Xavier Capó, Valentina Fagiano and Salud Deudero

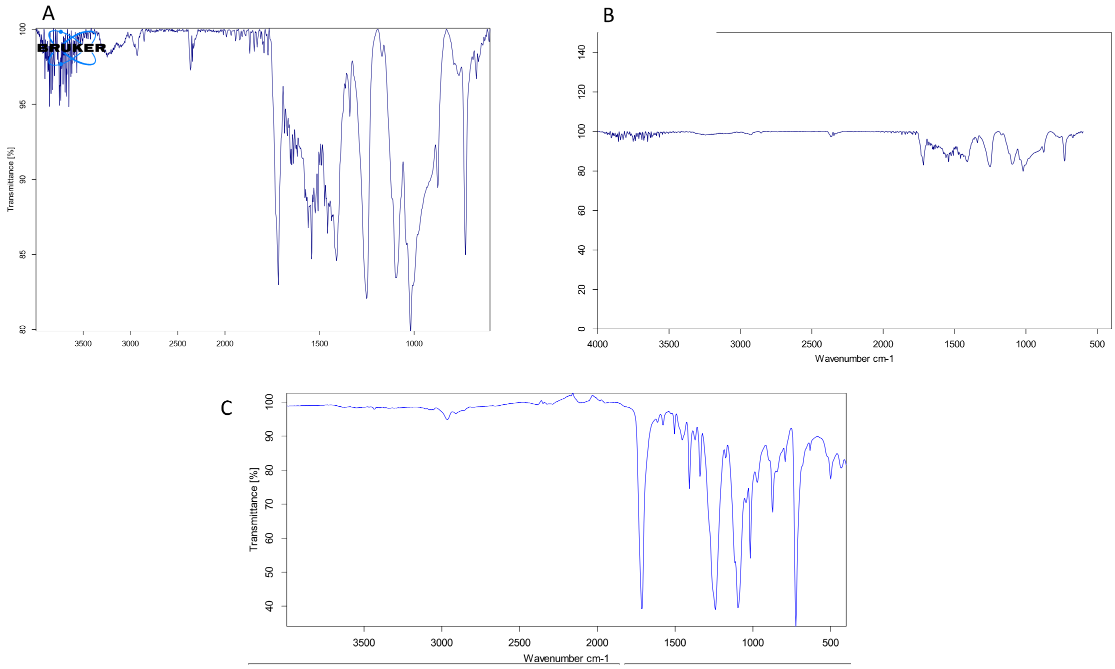

Figure S1. Example spectra for the following: (A) blue fiber of the material cellulose acetate found in an invertebrate (μFTIR), (B) blue fiber of the biodegradable material Ecoflex® found in a fish species (μFTIR) and (C) black fragment of polyethylene terephthalate found in a fish species (ATR-FTIR). All items had a quality hit > 70%.
